# Strategic Deception in Adults with Autism Spectrum Disorder

**DOI:** 10.1007/s10803-020-04525-0

**Published:** 2020-05-17

**Authors:** Bob van Tiel, Gaétane Deliens, Philippine Geelhand, Anke Murillo Oosterwijk, Mikhail Kissine

**Affiliations:** 1 Donders Institute for Brain, Cognition, and Behaviour, Radboud University Nijmegen, Kapittelweg 29, 6525 EN Nijmegen, The Netherlands; 2 Université libre de Bruxelles, Bruxelles, Belgium; 3 Rotterdam School of Management, Rotterdam, Netherlands

**Keywords:** Deception, Perspective-taking, Theory of mind, Autism, Strategy

## Abstract

Autism Spectrum Disorder (ASD) is often associated with impaired perspective-taking skills. Deception is an important indicator of perspective-taking, and therefore may be thought to pose difficulties to people with ASD (e.g., Baron-Cohen in J Child Psychol Psychiatry 3:1141–1155, 1992). To test this hypothesis, we asked participants with and without ASD to play a computerised deception game. We found that participants with ASD were equally likely—and in complex cases of deception even more likely—to deceive and detect deception, and learned deception at a faster rate. However, participants with ASD initially deceived less frequently, and were slower at detecting deception. These results suggest that people with ASD readily engage in deception but may do so through conscious and effortful reasoning about other people’s perspective.

## Introduction

We perceive and interpret the way other people behave in order to predict their upcoming actions and adjust our own behaviour accordingly (e.g., Dennett 1987; Premack and Woodruff 1978; Sellars 1956). Since it is common knowledge that people interpret each other’s actions in this way, the possibility of *strategic deception* (hence, simply, deception) emerges: we may intentionally carry out an action that is likely to be misinterpreted in order to obtain a strategic advantage (e.g., Goffman 1959; Harrington 2009).

It is sometimes assumed that deception necessarily involves *perspective-taking*, or, equivalently, having a *theory of mind* (e.g., Premack and Woodruff 1978; Ruffman et al. 1993). According to this idea, deceivers have to take into consideration how others will perceive and interpret their actions. Thus, e.g., a poker player may raise, i.e., increase the size of the bet, despite being dealt a bad hand of cards by reasoning that other players are likely to interpret the raise as signifying a good hand of cards—since presumably one would not voluntarily play for more money with a bad hand of cards. Hence, raising will increase the probability of the other players folding, i.e., dropping out of the game and losing the bet (Sklansky 2005). In line with this idea, the capacity of efficient deception seems to be highly correlated with complex social perspective-taking (e.g., Talwar and Gordon 2007).

However, deception can also be purely based on *social learning* of causes and effects (e.g., Byrne and Whiten 1991, 1992). By repeatedly interacting with others, one may observe regularities that can subsequently be exploited to predict how others will respond to one’s actions. In the case at hand, the poker player may simply associate raising with an increased probability of folding based on repeated interactions, without actually representing the perspective of the other players at all. Such socially learned strategies can serve as fast and frugal alternatives to perspective-taking, but also as a (compensatory) strategy for explicitly going through the normally implicit reasoning steps that underlie perspective-taking (e.g., Blokpoel et al. 2012; Dienes and Perner 1999).

Perspective-taking and social learning are distinct constructs, but they are also importantly interconnected. Thus, the development of perspective-taking in children is at least to some extent reliant on social learning, as shown, e.g., by impaired perspective-taking skills in children who have experienced severe social deprivation (e.g., Kreppner et al. 1999; Yagmurlu et al. 2005). Conversely, the ability to take another person’s perspective provides a scaffolding for streamlining social learning (Heyes and Frith 2014). As a consequence of this interconnectedness, certain instances of deceptive behaviour may be explained both in terms of perspective-taking and social learning.

In spite of this aetiological ambiguity, the ability to deceive—and correspondingly the ability to realise that one is being deceived—is often viewed as key evidence for the presence of perspective-taking abilities (e.g., Ruffman et al. 1993; Woodruff and Premack 1979). As Chandler et al. (1989, p. 1267) put it:

...any organisms that can be shown to actively distort or fabricate information in novel ways specifically intended to mislead others into accepting as true what they themselves know to be false deserve to have it said of them that they subscribe to some theory of mind.

Hence, it is predicted that agents with impaired perspective-taking abilities also show impairments in the ability to deceive or detect deception.

People with Autism Spectrum Disorder (ASD) are often seen as having impaired perspective-taking abilities, i.e., as being *mind-blind* (e.g., Baron-Cohen 1989; Baron-Cohen 1995; Frith and Happé 1994; Happé 1993). Early studies even took mind-blindness to be the defining characteristic of ASD (e.g., Happé 1993). However, more recent studies have emphasised the important individual differences in ASD which are unlikely to be reducible to a single deficit (Pellicano 2010; Vivanti et al. 2019). Indeed, several studies have shown that in suitable experimental settings high-functioning children and adults with ASD appear perfectly able to take other people’s perspectives (e.g., Begeer et al. 2010, 2003; Chevallier 2012). However, even these more nuanced studies acknowledge that difficulties with perspective-taking are widespread in people with ASD. Hence, people with ASD are predicted to have difficulties with deception and deception detection.

Experimental data from children with ASD broadly confirm this prediction (e.g., Baron-Cohen 1992; Dennis et al. 2000; Li et al. 2011; Oswald and Ollendick 1989; Russell et al. 1991; Sodian and Frith 1992; Yang et al. 2017). To illustrate, Baron-Cohen (1992) engaged 15 children with ASD (mean chronological age: 15.3, mean mental age: 6.2), 15 children with learning difficulties who were matched for chronological and mental age, and 15 unmatched but substantially younger typically developing children (mean chronological age: 3.8) in a penny-hiding game (Gratch 1964). The game consisted of two stages.

In the first stage, the experimenter repeatedly hid a penny in one of his hands, and children had to guess the location of the penny by pointing at one of the experimenter’s hands. Success at this simple game of deception detection required avoiding obvious response patterns which could easily be exploited by the experimenter. Four of the 15 children with ASD failed to do so, and consistently pointed at the same hand over and over again. All other children with and without ASD varied their choices across trials.

In the second stage, the children took on the role of penny-hider. In this stage, successful deception required concealing any information that the experimenter could exploit to guess the correct hand; e.g., not hiding the penny in plain sight and keeping both hands closed when the experimenter was choosing. 10 children with learning difficulties and 13 typically developing children succeeded at this form of information-occlusion. By contrast, only 2 of the children with ASD succeeded at hiding circumstantial evidence that allowed the experimenter to determine the location of the penny.

The results of this study—as well as all other studies on the topic (see references above)—indicate that many children with ASD have difficulties with deception. These difficulties are usually connected to their more general problems with perspective-taking; e.g., Baron-Cohen attributes these problems to the inability of children with ASD to “appreciate someone else’s mental states (such as their thoughts and beliefs), and to make sense of and predict their behaviour on the basis of such states” (p. 1141).

It is less clear, however, whether the difficulties with deception that children with ASD experience persist into adulthood; however, the limited evidence that has been collected so far suggests that they do. Thus, Happé (1994) found that adults with ASD provided less accurate descriptions of the intentions of story characters telling white lies or engaging in double bluffs (i.e., actions that are intended to appear as a bluff but are actually genuine). In a more recent study, Williams et al. (2018) found that adults with ASD were significantly less accurate at determining whether people in videotaped interactions were lying or not, when compared to adults without ASD.

However, in order to accurately detect deception in these studies, participants had to rely on their knowledge of implicit norms that govern social interactions. Thus, to determine that the character in one of Happé’s stories was telling a white lie, participants had to represent and integrate the social norm that one should always appear grateful when receiving a gift. Similarly, in the study of Williams and colleagues, participants had to detect subtle facial and linguistic cues to establish whether the people in the videotapes were lying.

It is well known that people with ASD have problems with this type of social reasoning (e.g., Baron-Cohen et al. 2001; Deliens et al. 2018; Golan et al. 2006). Hence, it could be that the observed difficulties that participants with ASD have with deception detection may be due, at least in part, to impaired knowledge of the norms that govern social interactions rather than difficulties with deception *per se*.

In summary, there is tentative evidence suggesting that adults with ASD have difficulties accurately detecting and understanding deceptive behaviour, but this evidence stands in need of confirmation in an experimental setting that does not rely on participants’ knowledge of implicit social norms. Moreover, the ability of adults with ASD to actively perpetrate deception has—to the best of our knowledge—not been studied at all. Hence, this paper reports on an experiment that explores the active and passive deceptive abilities of adults with ASD in an experiment that does not draw upon knowledge of social interactions.

### The Task

In order to measure participants’ ability to use and detect deception, we engaged them in a game against a computerised opponent (inspired by Yoshida et al. 2010). The deception game differed from previous studies in at least two important respects. First, the goal and rules of the game were made explicit, so that participants did not have to rely on implicit social norms to understand the opponent’s behaviour. Second, the behaviour of the opponent was fully manifest, so that participants did not have to (in fact, could not) attend to subtle cues belying the opponent’s intention. In these ways, then, the deception game did not require participants to represent and integrate any implicit social norms or cues.

An additional feature of the deception game is that people with ASD may experience less social anxiety when they play a computerised game than when they interact with another person, which seems especially pertinent given that the interaction involves deception (Bölte et al. 2002; Heiman et al. 1995). Conversely, people without ASD may perform at elevated levels in socialised settings because they are more sensitive than people with ASD to the audience effect, i.e., the desire to perform well to enhance their reputation in the eyes of the experimenter (e.g., Chevallier et al. 2014). For these reasons, we opted for an experimental setting of a less social nature, in that participants played against a computerised opponent and were presumably intrinsically motivated to obtain a high score rather than by the desire to please the experimenter.

**Fig. 1:**
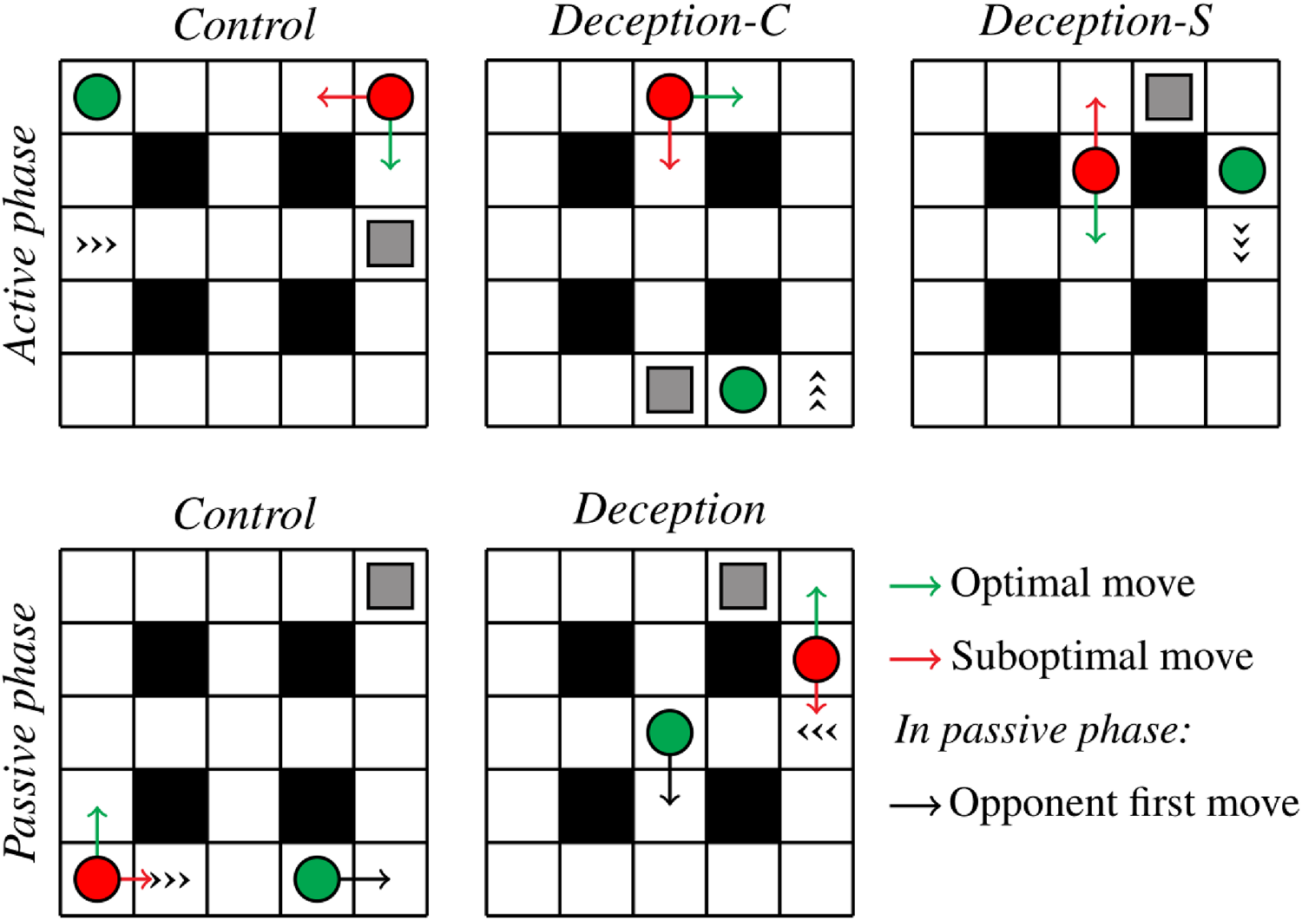
In the Active phase, the opponent (green circle) is unable to see the treasure (gray square) and the participant (red circle) moves first. Control trials do not involve any deception. In the Deception-C trial, the treasure can only be captured if the player deceives (i.e., moves right) to trap the opponent on the trap tile, which can only be escaped from in the direction of the arrowheads. In the Deception-S trial, the treasure can be captured by moving directly towards the treasure (i.e., up) or by first moving down to trap the opponent; but the latter option leads to a higher score. In the Passive phase, participants are unable to see the treasure and the opponent moves first (the black arrow). In Control trials, the opponent moves directly towards the treasure. In the Deception trial, the opponent first moves away from the treasure (i.e., down) to trap the opponent on the trap tile. In the Passive phase, trap avoidance was coded as optimal even in Control trials, in which it did not affect the outcome, because trap avoidance was a superior global strategy and participants had no way of telling apart the two trial types, given that they were unable to see the location of the treasure

During the game, participants controlled a red circle that could move around a 5 × 5 grid (see Fig. 1). The goal of the game was to capture a treasure. The grid also contained a computerised opponent. The opponent also tried to capture the treasure. The participant and the opponent took turns moving. Whoever captured the treasure first was awarded a number of points that depended on the distance between the treasure and the other player. Hence, participants’ goal was to capture the treasure first and maximise the distance between the treasure and the opponent. Crucially, the grid also contained a trap tile. The trap tile could be accessed from all sides, but could only be escaped from in one specific direction.

The experiment consisted of two stages. During the *Active* stage, participants were told that the opponent was unable to see the location of the treasure, and thus relied on the participants’ movements to guess where the treasure was located. Hence, on critical trials, participants could obtain a greater reward by deceptively first moving away from the treasure in order to trap the opponent on the trap tile. During the *Passive* stage, the player and opponent switched roles, and it was the player who was unable to see where the treasure was located. The opponent was programmed to routinely deceive the player by moving away from the treasure in order to trap the player on the trap tile. The optimal behaviour for the player was thus to consistently avoid entrapment on the trap tiles.

### Predictions

As noted in the introduction, one can engage in deception either by taking the perspective of the other person or by social learning of causes and effects, i.e., by exploiting regularities in behaviour that one may observe from repeated interactions.

In the context of the game, a perspective-taking player may reason as follows: if I move away from the treasure, the opponent will interpret that move as evidence that the treasure lies in that direction and move accordingly. Doing so will trap the opponent and thus allow me to capture the treasure. This type of perspective-taking is often thought to proceed relatively automatically and effortlessly (Schneider et al. 2017).

It has been argued that people with ASD may compensate for their hypothesised inability to engage in perspective-taking by means of social learning strategies (e.g., Dean et al. 2017; Griffin and Dennett 2008; Happé 1995; Livingston et al. 2019). To give an example, someone with ASD may compile a list of cues for jokes to make sure they laugh when their interlocutor makes a joke they do not understand (Livingston et al. 2019). Similarly, players in our experiment may engage in strategic deception by exploiting regularities that they distill from repeated interactions with their opponent. Crucially, behaving on the basis of socially learned regularities is usually thought to be cognitively taxing when compared to perspective-taking (e.g., Hull et al. 2017).

Hence, if we find that people with ASD are less likely to deceive or realise that they are being deceived, this would provide strong evidence for the mind-blindness tenet that people with ASD are unable to to take other people’s perspective. However, if we find that people with and without ASD are equally likely to engage in strategic deception, there are at least two possible explanations. First, it could constitute evidence against the mind-blindness theory of ASD. Second, as we just discussed, it could be that people with ASD compensate for their mind-blindness by using socially learned strategies.

Such compensatory strategies may be observed in our experiment in at least two ways. First, if people with ASD have to distill regularities from the experiment, we may expect that there is a stronger learning effect for people with ASD than for people without ASD, i.e., that people with ASD are initially less likely to deceive or realise they are being deceived, but that this difference diminishes over the course of the experiment. Second, if strategic deception involves conscious reasoning about these regularities, we may expect that the use and detection of deception is more time-consuming for people with ASD.

## Methods

### Participants

53 participants were recruited. 27 participants had received a prior clinical diagnosis of ASD (13 females, mean age: 33.7, standard deviation: 9.4); the remaining 26 participants had not (11 females, mean age: 29.4, standard deviation: 8.5). The proportions of males and females did not differ across the two groups (*χ*^2^ < 1). Participants with ASD were marginally older than participants without ASD (*t*(51) = 1.77, *p* = .08); however, we did not expect any relation between age and deception. The presence or absence of ASD was assessed and confirmed for all participants using the Autism Diagnostic Observation Schedule (ADOS; Lord et al. 2012), which was carried out by a research-accredited assessor.

Participants with ASD were recruited from the Autism in Context: Theory and Experiment (ACTE) register of volunteers. Participants without ASD were recruited through announcements placed on the internet. Study inclusion criteria included: (i) being between 18 and 60 years old, (ii) having a global IQ above 70, (iii) having normal or corrected-to-normal vision and hearing, and (iv) in the case of participants without ASD, not having any known psychiatric, developmental, or neurological disorders.

Participants’ IQ was measured using the Wechsler Adult Intelligence Scale (WAIS-IV; Wechsler 2008). There was no significant difference between the mean IQs of participants with ASD (mean IQ: 119, SD: 16) and without ASD (mean IQ: 116, SD: 12; *t*(51) = 0.8, *p* = .43). Furthermore, we measured participants’ Autism Quotient (AQ; Baron-Cohen et al. 2001) and Empathy Quotient (EQ; Baron-Cohen and Wheelwright 2004). As expected, the mean AQ was significantly higher for participants with ASD (mean AQ: 39, SD: 5) than without ASD (mean AQ: 14, SD: 6; *t*(50) = 16.8, *p* < .001); conversely, the mean EQ was significantly lower for participants with ASD (mean EQ: 20, SD: 9) than without ASD (mean EQ: 40, SD: 11; *t*(50) = − 71, *p* = .001). Table 1 provides an overview of the demographic properties of the participants.

**Table 1:** Participants’ demographic features. Standard deviations between brackets. M/F: Number of males and females. AQ: Autism Quotient. EQ: Empathy Quotient. Diff: Significance of the difference, as measured with chi-squared tests or *t*-tests

	M/F	Age		IQ		AQ		EQ	
ASD	14/13	34 (9)		119 (16)		39 (5)		20 (9)	
TD	15/11	29 (9)		116 (12)		14 (6)		40 (11)	
Diff	n.s.	.083		n.s.		< .001		< .001	

### Materials and Procedure

The deception game took place on a 5 × 5 grid (Fig. 1). Four of the tiles were inaccessible. The accessible tiles showed (i) a red circle representing the participant, (ii) a green circle representing the computerised opponent, and (iii) a gray square representing the treasure (only in the Active phase, see below).

Participants’ goal was to capture the treasure before their opponent did. If they succeeded, they received a number of points equivalent to the number of steps the opponent would need to reach the treasure. Conversely, if the opponent captured the treasure first, participants lost a number of points equivalent to the number of steps they would need to reach the treasure. One of the remaining accessible tiles functioned as a trap. The trap was represented by three arrowheads. The trap was accessible from all sides but could only be escaped from in the direction of the arrowheads.

The participant and the opponent took turns moving. Both the participant and the opponent could move to any accessible, adjacent tile that was either up, down, left, or right from the tile on which they were standing. Participants registered their movement by pressing the arrow keys on their keyboard, and had 10 s to make each move. If they failed to make a move within that time frame, they lost their turn and the opponent moved. This happened in less than 1% of the trials. The opponent moved immediately after the participant released the movement key, whereupon the participant again had 10 s to make the next move. Each round ended after either the participant or the opponent captured the treasure, or after the participant had made 10 movements without anyone capturing the treasure; however, the latter never occurred in the experiment.

The experiment consisted of two phases: the *Active* phase and the *Passive* phase. In the Active phase, the participant always moved first. Participants were told (through written instructions on the screen) that the opponent was unable to see where the treasure was, and therefore had to guess its location based on the participant’s behaviour. The opponent was programmed so that it always moved in the same general direction in which the participant was moving. The opponent thus moved in a way that was—at least in principle—completely predictable, and its behaviour was similar to how one might expect a naive person to behave.

The Active phase consisted of two types of trials. In *Control* trials, the optimal path was for participants to move directly towards the treasure. In some of these trials, this would result in the participant capturing the treasure; in others, it was inevitable that the opponent would capture the treasure. In *Deception* trials, the optimal path was to first move away from the treasure in order to trap the opponent using the arrow tiles. There were two types of deception trials: in *Deception-C* trials, moving directly towards the treasure would result in the opponent capturing the treasure. In *Deception-S* trials, participants could capture the treasure by either moving directly towards the treasure, or by first moving away from the treasure to trap the opponent. However, the second method yielded a greater payoff, since the opponent would be further away from the treasure than if the participant moved directly.

There were 7 Control trials and 14 Deception trials (7 Deception-C trials and 7 Deception-S trials). Hence, the Active phase consisted of 21 trials in total. The order of the trials was randomised for each participant.

In the Passive phase, the opponent always moved first. Participants were unable to see the treasure, and therefore had to guess its location based on the opponent’s behaviour. However, the opponent would routinely (in two-thirds of the trials) deceive participants by moving away from the treasure first in order to trap participants by means of the arrow tiles. The optimal behaviour from the participants’ perspective was thus to consistently avoid being trapped. On Control trials, this would lead to a slightly suboptimal outcome; on the more frequent Deception trials, however, trap avoidance would lead to a vastly superior outcome. Note that participants were unable to distinguish Control and Deception trials in the Passive phase, since they were unable to see the treasure. Hence, both trial types will be grouped together for the purpose of analysis.

Figure  1 shows example starting positions for both phases. In the example Control trial of the Active phase, participants win 5 points by immediately going down. In the example Deception-C trial, moving down results in the opponent moving left and thus capturing the treasure. In order to capture the treasure, participants have to first move right and then proceed towards the treasure. In the example Deception-S trial, moving up and then right yields 1 point. First moving down and then moving towards the treasure yields a score of 5. In the example Control trial of the Passive phase, the opponent moves right and then up twice. Irrespective of whether participants move up or right, they lose 4 points. In the example Deception trial, the opponent first moves down to trap the participant, and then moves up again to capture the treasure. In order to capture the treasure, participants have to move in the opposite direction from the opponent’s initial move.

Participants were first trained to move around the grid. Afterwards, they were familiarised with the movement of the opponent, and how the opponent’s moves depended on theirs. Then, the trap tiles were introduced, and participants played five training rounds to familiarise themselves with the workings of these trap tiles. Then, the treasure was introduced, and participants played five rounds to familiarise themselves with the scoring system. After that, the Active phase started, which was always followed by the Passive phase. We did not counterbalance the order of the two phases because we intuited that the Passive phase was considerably more challenging than the Active phase.

## Results

### Data Preparation

13 trials were removed because participants failed to move within 10 s (0.6% of the trials). In addition, 30 trials were removed because the responses failed to register (1.3%).

### Behaviour

**Fig. 2:**
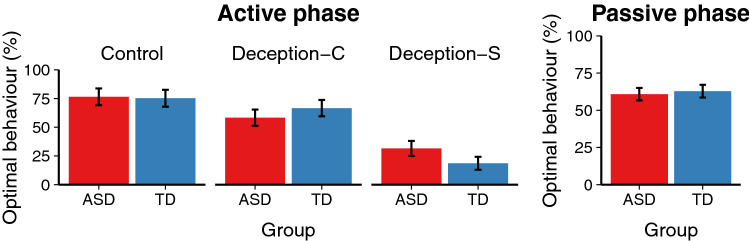
Frequency of optimal behaviour across trial types. Error bars represent 95% confidence intervals

Behaviour in the Active phase was coded as optimal or suboptimal. In Control trials, optimal behaviour was to move directly towards the treasure. In Deception trials, optimal behaviour was to first move away from the treasure in order to trap the opponent. Figure 2 shows the mean percentages of optimal behaviour for participants with and without ASD.

To determine whether people with and without ASD behaved differently, we constructed generalised binomial mixed effects models predicting optimal behaviour based on diagnosis (ASD or TD), trial number, age, and IQ, including random slopes and intercepts for participants and items (Barr et al. 2013). For Control trials and Deception-C trials, there were no significant effects of diagnosis (both *Z*’s < 1). For Deception-S trials, however, participants with ASD were significantly *more* likely to behave optimally than participants without ASD ($\beta = -1.69, SE = 0.74, Z = -2.29, p = .022$).

Behaviour in the Passive phase was also coded as optimal or suboptimal. Optimal behaviour was to avoid getting trapped on the arrow tiles by moving in a different direction from the opponent. Participants without ASD were slightly more likely to behave optimally than participants with ASD (63% vs. 61%). In order to determine if this difference was significant, we constructed a generalised binomial mixed effects model predicting optimal behaviour based on diagnosis, trial number, age, and IQ, including random slopes and intercept for participants and items. There was no significant effect of diagnosis ($Z < 1$).

One of our reviewers asked for an indication of the sensitivity of our task, i.e., given the number of participants and items, differences of which magnitude could likely be detected in our experiment? To answer this question, we calculated 95% confidence intervals around the parameter estimates (Baguley 2009; Levine and Ensom 2012). The confidence interval indicates a range in which the population mean (i.e., the genuine effect of ASD) is 95% likely to occur. In the Active phase, the confidence interval lies between −0.02 (i.e., people with ASD are 2% more likely to deceive) and 0.11 (i.e., people with ASD are 11% less likely to deceive). In the Passive phase, the confidence interval lies between −0.02 (i.e., people with ASD are 2% more likely to deceive) and 0.08 (i.e., people with ASD are 8% less likely to deceive).

### Learning

**Fig. 3:**
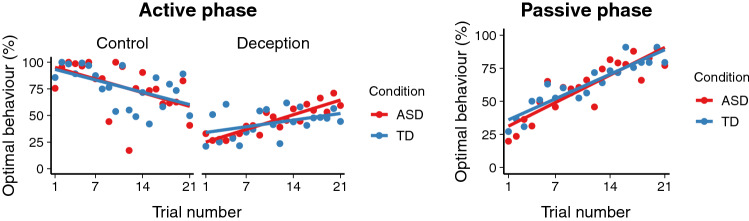
Frequency of optimal behaviour plotted against trial number

Figure  3 shows the mean percentages of optimal behaviour for each trial number.

In order to determine whether participants with and without ASD learned to deceive at different rates in the Active phase, we constructed generalised binomial mixed effects models predicting optimal behaviour on the basis of diagnosis (ASD or TD), trial number, their interaction, age, and IQ, including random slopes and intercepts for participants, and random intercepts for items, which was the maximal converging model.

Figure 3 visually suggests that performance on Control trials becomes worse throughout the experiment. However, we observed no significant effect of trial number in Control trials ($\beta = 0.05, SE = 0.05, t = 1.05, p = .30$). A possible explanation for the non-significant downward trend may be that as participants became more likely to deceive throughout the experiment (see the next paragraph), they also became more likely to misapply the deceptive strategy to trials where it was suboptimal. We also observed no significant effects of diagnosis or its interaction with trial number on Control trials (both *Z*’s $< 1$).

For the two types of Deception trials, there was a significant interaction between diagnosis and trial number ($\beta = -0.10, SE = 0.04, Z = -2.35, p = .019$), and a significant effect of trial number ($\beta = 0.17, SE = 0.04, Z = 4.08, p < .001$), but no significant effect of diagnosis ($\beta = 1.16, SE = 0.72, Z = 1.62, p = .11$). The significant interaction indicates that, on trials that suggested the use of strategic deception, participants with ASD improved to a greater extent throughout the experiment than participants without ASD. In visual terms, Fig. 3 thus shows that, in the Deception condition, the red line for participants with ASD starts below the blue line for participants without ASD, but increases more steeply.

In order to locate the source of the interaction effect, we also analysed the probability of deception in the first and final third of the experiment separately. For the first third of the experiment, we observed a significant effect of diagnosis ($\beta = 1.03, SE = 0.50, Z = 2.05, p = .04$), so that participants without ASD were more likely to deceive than participants with ASD. No significant difference between the two groups was observed in the final third of the experiment ($\beta = -0.46, SE = 0.45, Z = -1.01, p = .31$).

In order to determine whether participants with and without ASD learned to detect deception at different rates in the Passive phase, we constructed a generalised binomial mixed effects model predicting optimal behaviour on the basis of diagnosis (ASD or TD), trial number, their interaction, age, and IQ, including random slopes and intercepts for participants and items. There were no significant effects of diagnosis or its interaction with trial number (both *Z*’s $< 1$). However, there was a significant effect of trial number ($\beta = 0.19, SE = 0.04, Z = 5.33, p < .001$).

### Response times

**Fig. 4:**
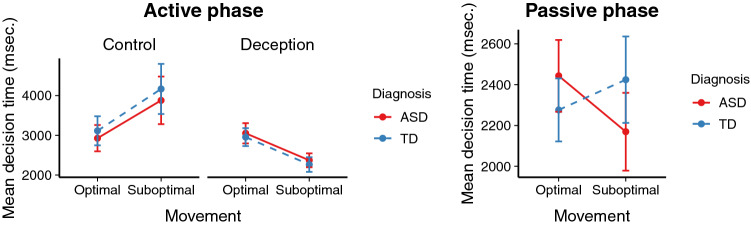
Response times for optimal and suboptimal movements. Error bars represent 95% confidence intervals

Figure  4 shows the mean response times for optimal and suboptimal movements.

In the Active phase, the first movement always indicated whether participants engaged in strategic deception or not. In order to analyse response times for these first movements in the Active phase, we constructed linear regression mixed effects models predicting logarithmised response times based on diagnosis (ASD or TD), behaviour (optimal or suboptimal), their interaction, trial number, age, and IQ, including random intercepts for participants and items, which was the maximally converging model.

For the Control trials, there were no significant effects of diagnosis or its interaction with behaviour (both *t*’s $< 1$). However, there was a marginally significant effect of behaviour ($\beta = -0.16, SE = 0.10, t = -1.70, p = .09$), indicating marginally slower responses when participants behaved suboptimally. For the trials involving deception, there were also no significant effects of diagnosis or its interaction with behaviour (both *t*’s $< 1$). However, there was a significant effect of behaviour ($\beta = -0.30, SE = 0.05, t = 5.78, p < .001$), such that participants were significantly slower when they behaved optimally, i.e., when they engaged in strategic deception than when they did not.

For the Passive phase, we concentrate on those moves that indicate that participants avoided entrapment. In most cases, this was the first move that participants made, but it could also be the second or third move if the arrow tile was further away from the participant’s starting position. To analyse response times for these moves, we constructed a linear regression mixed effects models predicting logarithmised response times based on diagnosis (ASD or TD), behaviour (optimal or suboptimal), their interaction, trial number, age, and IQ, including random intercepts for participants and items.

There was a significant interaction between behaviour and diagnosis ($\beta = -0.26, SE = 0.09, t = -2.84, p = .005$), and a significant effect of behaviour ($\beta = -0.32, SE = 0.07, t = 4.76, p < .001$), but no significant effect of diagnosis ($\beta = 0.22, SE = 0.14, t = 1.57, p = .12$). The significant interaction indicates that participants with ASD showed a different response time pattern than participants without ASD. In visual terms, Fig.  4 thus shows a crossed interaction effect between behaviour and diagnosis.

In order to locate the source of the interaction, we carried out follow-up analyses for each participant group separately. These analyses indicate that, whereas participants without ASD were equally fast in their optimal and suboptimal behaviour (*t* $< 1$), participants with ASD were significantly slower when they behaved optimally, i.e., when they avoided entrapment on an arrow tile ($t = 0.36, SE = 0.08, t = 4.47, p < .001$). In other words, participants with ASD were slowed down when they had to override the tendency to move in the same direction as their opponent because they thought they were being deceived.

This analysis also suggests that the significant main effect of behaviour is mostly due to the slowdown of participants with ASD when behaving optimally, and does not indicate an overall slowdown for optimal behaviour.

## Discussion

It is often assumed that strategic deception requires taking another person’s epistemic perspective. Hence, given that people with ASD are thought to have important difficulties with perspective-taking (e.g., Baron-Cohen 1995), one might expect that people with ASD also experience difficulties with strategic deception. Previous studies seem to confirm this hypothesis (Happé 1994; Williams et al. 2018). Both of these studies, however, relied on participants’ knowledge of social norms, which may have confounded the results.

This study measured the deceptive abilities of people with and without ASD in an experimental setting where the social demands were less stringent. Participants played a game against a computerised opponent in which either the opponent (Active phase) or the participants themselves (Passive phase) were unable to see the location of a treasure that had to be captured. Consequently, in the Active phase, one could deceive by steering the opponent onto one of the trap tiles that forced the opponent further away from the treasure. In Deception-C trials, deception was a prerequisite for capturing the treasure; in Deception-S trials, deception merely led to a higher score. In the Passive phase, the opponent would routinely attempt to deceive the participant, and participants had to avoid entrapment—and hence avoid being deceived—in order to reach an optimal outcome.

We found that participants with and without ASD were equally likely to deceive in Deception-C trials and equally likely to realise that they were being deceived during the Passive phase. In Deception-S trials, participants with ASD were even *more* likely to deceive than participants without ASD. An analysis of the confidence intervals around the parameter estimates indicated that the population difference between people with and without ASD was almost certain to be less than 10%. Even if a more highly-powered study is able to detect such subtle differences, they nevertheless appear to challenge the idea that ASD is characterised by an impairment in the ability to take other people’s perspective.

However, an alternative explanation for the absence of an effect of ASD is that participants with ASD used socially learned strategies to compensate for their difficulties with perspective-taking, i.e., they derived regularities from observed behaviour and reasoned about those regularities to mimic perspective-taking (e.g., Livingston et al. 2019). Crucially, the use of such socially learned strategies is thought to be more deliberate and cumbersome than perspective-taking in people without ASD (e.g., Dean et al. 2017; Hull et al. 2017).

The distinction between perspective-taking and socially learned strategies has also been invoked in studies on false-belief reasoning. In the standard false-belief task, participants watch a character place, e.g., a marble in a basket. Unbeknownst to the character, the marble is moved to a different location (Wimmer and Perner 1983). Afterwards, participants are asked where they think the character will look for the marble, which requires them to take the perspective of the character. High-functioning people with ASD mostly give the correct answer to the false-belief question, even though they start doing so at a significantly older age than people without ASD (e.g., Happé 1995).

However, various studies suggest that people with ASD who pass the false-belief task use a different, less automatic and more cumbersome strategy than people without ASD to solve the task (e.g., Begeer et al. 2003; Senju 2012; Sommer et al. 2018). For example, eye-tracking research shows that, when watching the false-belief story unfold, people with ASD tend to look at the location where the marble is, whereas people without ASD tend to look at the location where the character thinks the marble is (Senju 2012). Similarly, fMRI research shows that people with ASD recruit a broader network of brain areas to solve the false-belief task, suggesting increased cognitive demands (Sommer et al. 2018). As in the case of deception, then, the literature shows that people with and without ASD may solve the false-belief task in different ways: either by automatic perspective-taking or by a conscious and effortful compensatory strategy.

The results of our experiment provide two pieces of evidence suggesting that participants with ASD adopted socially learned strategies. First, participants with ASD showed a significantly stronger learning effect, such that they were initially less likely to deceive, but became equally likely to deceive towards the end of the game. Moreover, people with ASD were better able to transfer their deceptive abilities among different deception-consequences than people without ASD: from urgent instances in which deception was a precondition for obtaining the treasure to less urgent instances in which deception merely led to a higher score. Second, it took participants with ASD significantly longer to realise that they were being deceived when compared to participants without ASD. That is, in the Passive phase, participants were slower to move when they realised that they were being deceived.

Based on these observations, it may plausibly be argued that participants with ASD (successfully) used a different type of strategy to pass the experiment, i.e., a strategy that involves conscious and effortful reasoning about the regularities that they observed throughout the experiment. Notice, however, that these compensatory strategies were only partially visible: the increased learning effect was only observed when participants actively deceived; the increased response times when participants had to detect deception.

This study highlights the importance of distinguishing theoretically and empirically different ways in which people may engage in deception: either through perspective-taking or through social learning of causes and effects. This aetiological ambiguity generally makes it difficult to draw firm conclusions from the ability to strategically deceive to the ability to engage in perspective-taking (e.g., Byrne and Whiten 1991, 1992; Dennett 1978). However, we have shown that a more careful analysis of learning patterns and response times may distinguish the various mechanisms that underlie deception.

One may worry that, since participants played against a computerised opponent, deception in this particular setting did not draw upon perspective-taking abilities. Indeed, while people without ASD readily attribute beliefs and intentions to inanimate objects (e.g., Gergely and Csibra 2003), people with ASD have been found to do so less frequently (Castelli et al. 2002) or at least less successfully (Abell et al. 2000). At the same time, however, it has been found that people with ASD perform comparably in computerised and non-computerised versions of the false-belief task, suggesting that they are equally likely to engage in perspective-taking in both modalities. In addition, people with and without ASD may behave differently in more socially embedded experimental settings, e.g., because of people with ASD experiencing social anxiety or people without ASD being more motivated to perform well in such contexts (e.g., Chevallier et al. 2014). Hence, our computerised game may have offered a more neutral testing ground to compare the deceptive abilities of people with and without ASD.

Another concern is to extend our results to the deceptive abilities that individuals with ASD may display in real-life situations. Unlike the well-defined confines of our computerised game, real-life deception involves a complex and indeterminate interplay of social norms and verbal and non-verbal behaviour (e.g., Dennis et al. 2000; Ekman 1985; Vrij et al. 2000). It is well known that people with ASD have problems interpreting such norms and cues (e.g., Baron-Cohen et al. 1999, 1997). Therefore our results should not be construed as implying that people with and without ASD are equally likely to deceive across the board—as indeed the studies of Happé (1994) and Williams et al. (2018) have already provided evidence against.

While it is incontrovertible that deception in everyday life heavily draws upon knowledge of social interactions, deception *per se* is a fundamental cognitive ability whose presence or absence is a matter of considerable theoretical interest, which is ultimately divorced from the question of how successfully one can make use of deception in social interactions. We have shown that, at least in principle, people with ASD are equally good—and in some cases even better—at deceiving and detecting deception than people without ASD. Thus, our study suggests that the problems that people with ASD experience with deception are likely due to their problems with the representation of social information rather than to a deficit in the ability to strategically deceive.

Of course, it remains an open question whether people with ASD would perform similarly when they would engage with people instead of computerised opponents—we aim to address this question in future research. What the results of the current study convincingly show, however, is that people with and without ASD are equally well-versed in the logic of deception (to borrow a phrase from one of our reviewers), at least in settings that do not draw upon rich knowledge of social interaction.
